# Enhancing intraoperative tumor delineation with multispectral short-wave infrared fluorescence imaging and machine learning

**DOI:** 10.1117/1.JBO.28.9.094804

**Published:** 2023-03-27

**Authors:** Dale J. Waterhouse, Laura Privitera, John Anderson, Danail Stoyanov, Stefano Giuliani

**Affiliations:** a University College London, Wellcome, EPSRC Centre for Interventional and Surgical Sciences, London, United Kingdom; b UCL Great Ormond Street Institute of Child Health, Cancer Section, Developmental Biology and Cancer Programme, London, United Kingdom; c Great Ormond Street Hospital for Children NHS Trust, Department of Specialist Neonatal and Paediatric Surgery, London, United Kingdom

**Keywords:** short-wave infrared, fluorescence-guided surgery, multispectral, machine-learning, cancer, neuroblastoma

## Abstract

**Significance:**

Fluorescence-guided surgery (FGS) provides specific real-time visualization of tumors, but intensity-based measurement of fluorescence is prone to errors. Multispectral imaging (MSI) in the short-wave infrared (SWIR) has the potential to improve tumor delineation by enabling machine-learning classification of pixels based on their spectral characteristics.

**Aim:**

Determine whether MSI can be applied to FGS and combined with machine learning to provide a robust method for tumor visualization.

**Approach:**

A multispectral SWIR fluorescence imaging device capable of collecting data from six spectral filters was constructed and deployed on neuroblastoma (NB) subcutaneous xenografts (n=6) after the injection of a NB-specific NIR-I fluorescent probe (Dinutuximab-IRDye800). We constructed image cubes representing fluorescence collected from ∼850 to 1450 nm and compared the performance of seven learning-based methods for pixel-by-pixel classification, including linear discriminant analysis, k-nearest neighbor classification, and a neural network.

**Results:**

The spectra of tumor and non-tumor tissue were subtly different and conserved between individuals. In classification, a combine principal component analysis and k-nearest-neighbor approach with area under curve normalization performed best, achieving 97.5% per-pixel classification accuracy (97.1%, 93.5%, and 99.2% for tumor, non-tumor tissue and background, respectively).

**Conclusions:**

The development of dozens of new imaging agents provides a timely opportunity for multispectral SWIR imaging to revolutionize next-generation FGS.

## 1 Introduction

Despite significant improvements in diagnosis and treatment, cancer remains the second leading cause of death worldwide (9.6  million/year).^1^ Surgical removal of the tumor is used in 45% of cancer treatments.^2^ Fluorescence-guided surgery (FGS) provides real-time visualization of tumors with molecular specificity by targeting tumor-associated molecules with fluorescently labeled molecular probes.^3^^–^^6^ The high-contrast delineation of tumor margins facilitates complete tumor resection whilst helping to preserve healthy surrounding structures.

Typically, FGS uses near-infrared (NIR) dyes emitting in the first biological window (NIR-I, 700 to 950 nm), where tissue shows diminished autofluorescence compared to visible-light wavelengths, enabling higher target-to-background ratios. Additionally, at these wavelengths, tissue is relatively transparent due to decreased absorption and scattering from hemoglobins, allowing deeper tissue penetration and visualization of sub-surface structures.^7^

In the second biological window (NIR-II, 1000–1350 nm), also known as the short-wave infrared (SWIR),^8^ autofluorescence, absorption, and scattering are further reduced. Still, interest in this region has been limited due to the limited SWIR fluorescence emitted from commercially available NIR-I dyes. However, recent work revealed this to be a consequence of the reduced sensitivity of traditional silicon detectors at SWIR wavelengths.^9^^,^^10^ Using InGaAs detectors revealed long SWIR emission tails of NIR-I dyes, opening the possibility of repurposing these NIR-I dyes for SWIR fluorescence imaging.^11^^,^^12^ This opportunity has sparked renewed interest in SWIR fluorescence imaging. Further driven by the decreased cost and increased availability of InGaAs sensors, interest in SWIR imaging has grown rapidly in recent years; for imaging ICG,^9^^,^^13^ for imaging small fluorescent molecules,^14^^–^^17^ for label-free imaging,^18^^–^^20^ and for its depth penetration.^21^^,^^22^

**Fig. 1:**
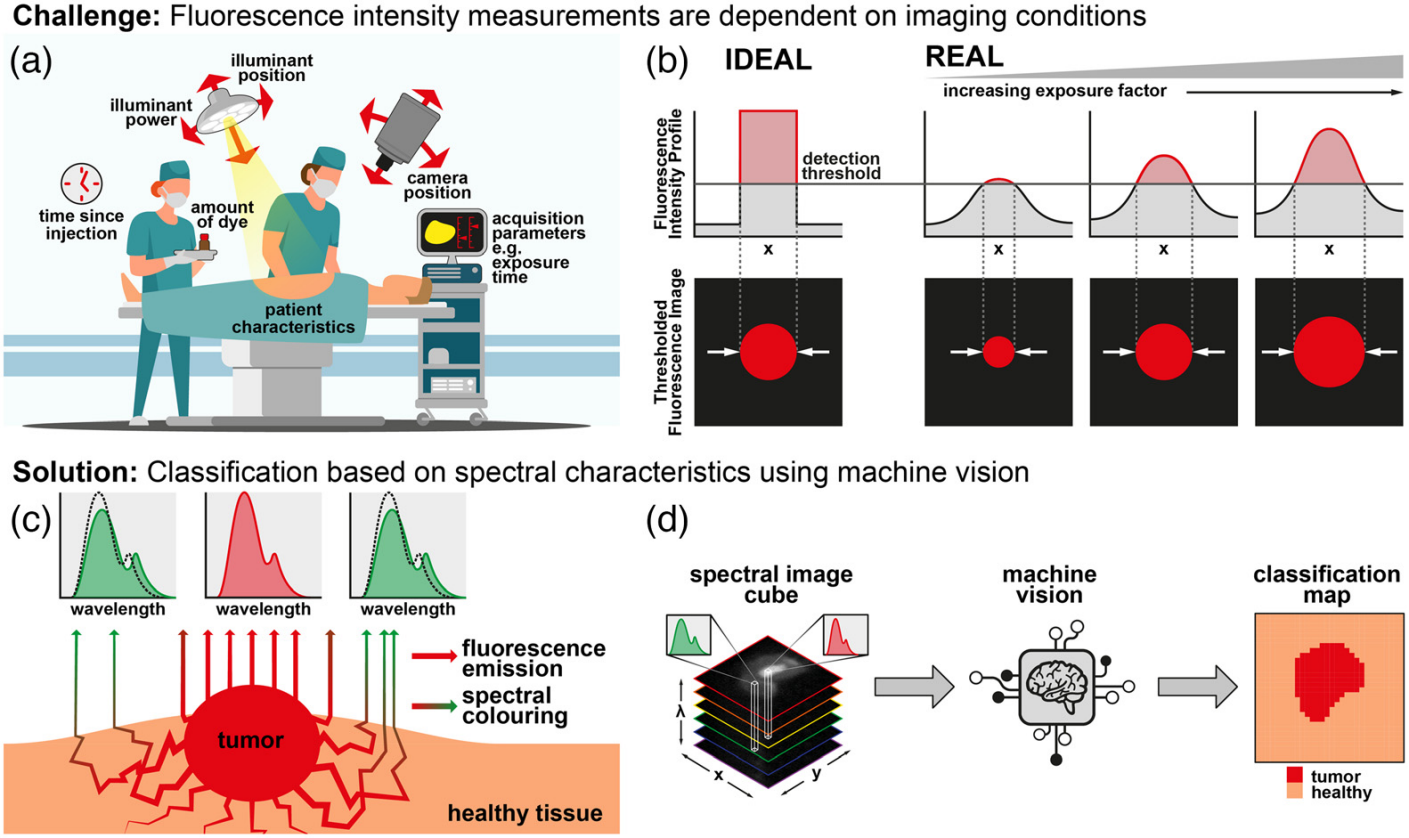
Rationale for the present study. (a) Schematic of the main external factors that can result in a multiplicative change of the measured fluorescence signal (exposure factors). (b) Thresholds are often applied to define tumor versus non-tumor. Visual representation of how changes in exposure factors can lead to over- or under-estimation of the extent of tumor tissue, leading to false positives and false negatives respectively. (c) The interactions of “pure” tumor fluorescence with surrounding tissue, which ultimately results in spectral coloring. (d) Schematic of the proposed solution. Spectral information captured in fluorescence imaging cubes (left) can be utilized by machine vision algorithms (center) to generate a classification map that can more accurately delineates the tumor region (right).

Still, FGS faces several challenges. During FGS, external factors affect the magnitude of the measured fluorescence signal [Fig. 1(a)]. Examples include the camera position and its exposure time, the illuminant position and its power, and the amount of dye found within the tumor (which is often related to the time since injection). Each of these factors results in a multiplicative change in the measured signal, termed the “exposure factor,” throughout this work.

Since the measured signal is a continuous function, thresholds are often applied to segment the image into tumor versus non-tumor regions—but since the signal profile across a tumor is not an ideal top-hat function, changes in exposure factor can result in misleading representations of the tumor, leading to over- or under-estimation of the extent of tumor tissue, and false positives/false negatives, respectively [Fig. 1(b)].

One way to overcome this challenge is to quantify luminous intensity (e.g., in mW/rad), such that a threshold can be defined independently of imaging conditions (e.g., >X mW/rad), but this is difficult as it requires careful calibration of devices, and the effects of tissue attenuation are not accounted for. A further, and more challenging step, is to remove the effects of light-tissue interaction altogether and to quantify the underlying fluorophore abundance (e.g., in moles) or concentration (e.g., in $\mathrm{mol}/{\mathrm{cm}}^{3}$). This requires hard work to compensate for many complex variables, including illumination non-uniformity, tissue absorption and scattering, and non-uniform light fluence. Though this is feasible in known and controlled imaging conditions, such as in a small animal preclinical imaging system, this quantification would be extremely challenging in a dynamic clinical environment.

We propose segmentation based on spectral characteristics of the measured fluorescence. As “pure” fluorescence light leaves the tumor, it interacts with absorbers and scatterers in the surrounding tissue, resulting in spectral coloring [Fig. 1(c)]—pure tumor fluorescence thus shows subtly different spectral characteristics to spectrally colored fluorescence arriving at the detector indirectly from surrounding tissue—allowing these two regions to be distinguished based on spectral characteristics of the detected light.

Spectral characteristics can be interrogated using multispectral imaging (MSI), an approach that captures spatially resolved (x,y) and spectral (wavelength, λ) information in a single “image cube” (x,y,λ). Typically, MSI was not useful for fluorescence imaging since the emission spectrum of most dyes spans only a single band on a typical multispectral imaging device (∼50  nm). However, the recently reported SWIR tail of NIR-I fluorophores^9^^,^^10^ covers several hundred nanometers, presenting an opportunity to measure the emission spectrum with multispectral imaging. Using machine-learning techniques, pure emission spectra can be distinguished from those scattered toward the camera by surrounding tissue, allowing a classification map to be generated [Fig. 1(d)].

Using this approach, we sought to develop a robust exposure-factor-independent method of visualizing tumor tissue during FGS. We created a custom multispectral SWIR fluorescence imaging device and undertook a preclinical imaging study to acquire *in vivo* multispectral fluorescence image cubes. These data were then subjected to machine-learning-based classification methods, indicating that multispectral SWIR fluorescence imaging has the potential to resolve tumor and non-tumor tissue with high accuracy during FGS.

## 2 Materials and Methods

### 2.1 Multispectral SWIR Fluorescence Imaging Device for Fluorescence-Guided Surgery

**Fig. 2:**
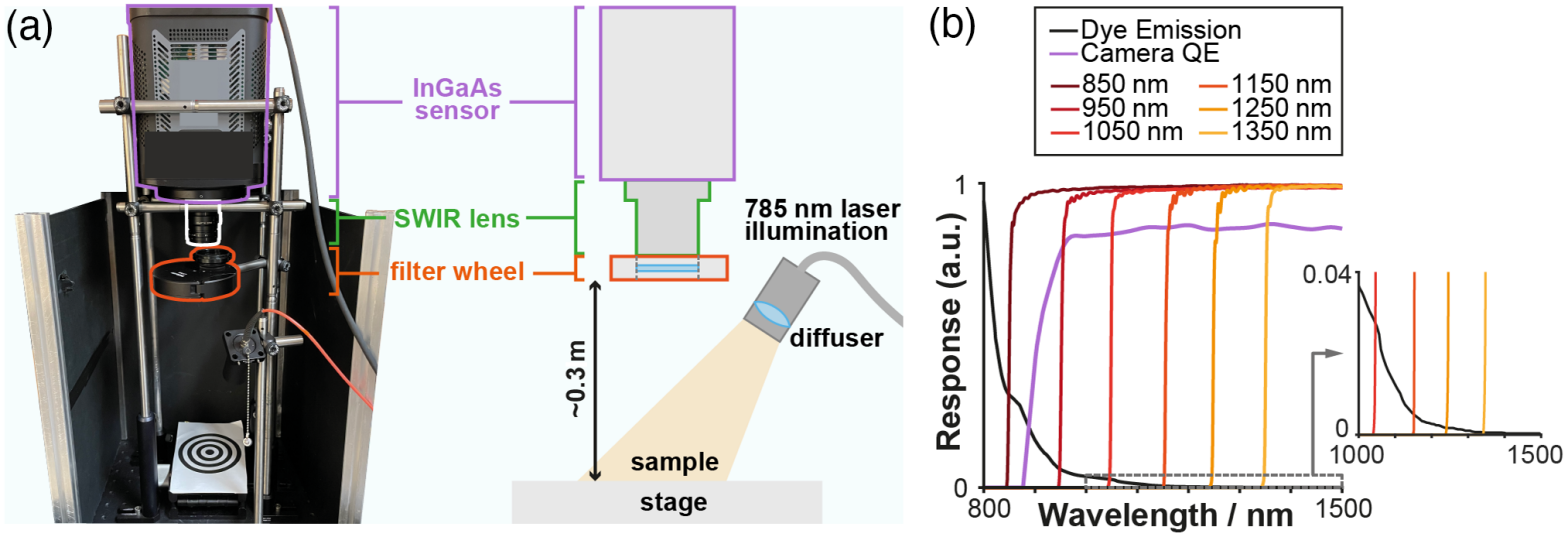
Multispectral NIR-I/SWIR fluorescence imaging device. (a) Photograph of the custom multispectral NIR-I/SWIR fluorescence imaging system alongside its schematic representation. (b) The spectral characteristics of the device are shown, including the QE of the sensor (purple line, data from Teledyne NIRvana specifications), the emission spectrum of IRDye800CW (adapted from Refs. 10 and 23, black line), and the transmission for the long pass filters (orange lines, data from Thorlabs FELH series specifications).

A multispectral SWIR fluorescence imaging system was designed and constructed (Fig. 2). Briefly, tissue is illuminated by a 785-nm fiber-coupled laser (BWF-1-785/55371, B&W Tek) dispersed onto the sample using a ground glass diffuser (DG10-220-MD, Thorlabs, Germany). SWIR fluorescence emission from the sample is collected by a highly sensitive InGaAs camera [QE>80% 950 to 1600 nm, NIRvana 640, Teledyne Princeton Instruments Fig. 2(b)] coupled to a SWIR lens (f=16  mm, F/1.4, Navitar, Canada). Fluorescence light is sequentially filtered using a six-position filter wheel (LTFW6, Thorlabs, Germany) through six long-pass filters with cut-off wavelengths of 850, 950, 1050, 1150, 1250, and 1350 nm [FELH series, Thorlabs, Germany, Fig. 2(b)]. The system was mounted inside a light-tight enclosure to remove background light. The camera was cooled to −80°C to reduce thermal noise.

### 2.2 In Vivo Fluorescence Imaging in a Small Animal Tumor Xenograft Model

This study assessed multispectral SWIR FGS in a subcutaneous animal model of neuroblastoma (NB). NB is an aggressive extracranial solid tumor accounting for 8% to 10% of all childhood malignancies and ∼15% of all cancer-related deaths in the pediatric population.^24^ With at least one-third of patients presenting with metastases at diagnosis, NB is one of the most challenging malignancies for pediatric oncologists and surgeons.^25^ Surgical resection of NB is challenging due to the localization, heterogeneity, and aggressive behavior of the tumor, compounded with the lack of real-time tools able to distinguish malignant tissue from the surrounding healthy tissue. The introduction of FGS in NB would transform surgery by providing an objective, real-time tool to visualize the extent of tumor resection, identify residuals and reliably assess the impact of surgical resection.

The recently developed molecular imaging probe Dinutuximab-IRDye800 was used in this study.^26^ Dinutuximab-beta (Qarziba), a clinically used monoclonal antibody, is targeted to the disialoganglioside antigen GD2 receptor, a clinically relevant tumor-associated antigen abundantly and ubiquitously expressed on almost all neuroblastic tumors, regardless of tumor stage.^27^^,^^28^ Dinutuximab-beta was conjugated to IRDye800CW (LI-COR Biosciences), the most used fluorophore conjugated to clinically approved monoclonal antibodies in clinical trials.^3^ The resulting conjugate will be referred to as “Dinutuximab-IRDye800” throughout the manuscript.

The performance of multispectral NIR-I/SWIR fluorescence imaging was assessed *in vivo* on a NB subcutaneous mouse xenograft. All experimental animal procedures were approved by the department of biological services and were carried out following local and international regulations. Briefly, human NB cells (LAN-1 cells, $2\times 10^{6}$) resuspended in Matrigel (100  μl, Appleton Woods Ltd, United Kingdom) were injected subcutaneously on the right flank of 6- to 8-week-old athymic nude female mice (CD1-Foxn1nu, Charles River Laboratories). Tumor growth was subsequently measured by calipers. Mice were intravenously injected with 100  μg (resuspended in 100  μl of PBS) of Dinutuximab-IRDye800 when the tumor was of an adequate size (∼5×6  mm, time t=0). At times t=24, 48, 72, and 96 h after injection, one mouse was euthanized, the tumor was exposed, and images were captured using the multispectral NIR-I/SWIR fluorescence imaging device. Two tumor-bearing mice not injected with the dye (negative control) were culled when the tumors reached a humane endpoint, and images were captured using the multispectral NIR-I/SWIR fluorescence imaging device.

### 2.3 Spectral Modeling

The published emission spectrum of IRDye800CW is known to be suppressed in the high wavelength region due to the low sensitivity of silicon sensors. The true emission spectrum of IRDye800CW was predicted by reflecting the data book absorption spectrum of IRDye800CW using the Franck–Condon principle. This was manually matched to the SWIR emission spectrum of IRDye800CW measured by Antaris et al.^10^ to predict a complete IRDye800CW emission spectrum (Fig. S1 in the Supplementary Material). This spectrum was propagated through the transmission characteristics of the system to predict the measured multispectral image spectrum according to

$$S_{\text{filter}}=\int T_{\text{filter}}(\lambda )\cdot QE(\lambda )\cdot IR800_{em.}(\lambda )\;d\lambda ,$$ (1)

where $T_{\text{filter}}(\lambda )$ is the data book transmission of each long pass filter, QE(λ) is the quantum efficiency (QE) of the camera, and $IR800_{em.}(\lambda )$ is the emission spectrum of IRDye800CW.

### 2.4 Image Processing

#### 2.4.1 Acquiring multispectral fluorescence image cubes

Image processing was performed in MATLAB (2022a, MathWorks). For each filter, images were captured using LightField^®^ (Teledyne Princeton Instruments) and saved as 16-bit TIFs for analysis. Images were captured at a range of exposure times (10 ms to 5 s) to ensure adequate signal without saturation. Raw images were checked for saturation (pixel values >35,000), and images with the highest exposure and no (or the least) saturated pixels were retained for analysis. A dark image was subtracted, and the image was normalized for exposure. The image from each filter was added to a final image cube (640  pixels×512  pixels×6 filters).

Each pixel in the image cube represents a six-element spectrum. These spectra were normalized using four different approaches: no normalization; max normalization (division by the maximum value in the spectrum); area under the curve (AUC) =1 (division by the sum of the 6-elements, equivalent to L1 vector normalization); and standard normal variate (SNV) normalization (subtracting a spectrum’s mean and dividing by its standard deviation).

For visualization, images captured using consecutive filters were subtracted to create a “band image” containing signal from a narrow band of wavelengths. For example, a 900-nm band image was produced by subtracting the image captured with a 950-nm long-pass filter from the image captured with an 850-nm long-pass filter, and thus contained signal from the range 850 to 950 nm.

#### 2.4.2 Defining regions of interest for each class

Regions of interest (ROIs) were drawn on each image to define: (1) the tumor; (2) a region of non-tumor tissue, taking care to avoid the areas around the liver and femur, where the off-target signal was apparent; and (3) a region of background from outside the animal. The ROIs were used to mask the images, defining pixels in each of the three classes: tumor, non-tumor tissue and background.

#### 2.4.3 Extracting fluorescence line profiles

To investigate the relationship between wavelength and the sharpness of tumor margins, line profiles across the tumor region were calculated. Lines were manually drawn across the tumor within the image. The image was rotated to orient this line horizontally. The line was then used to automatically select a rectangular ROI with a width of 3 pixels, thus defining three adjacent line profiles, which were subsequently averaged to define a final line profile across the tumor.

### 2.5 Classification of Multispectral Image Cubes to Discriminate Tumor and Non-Tumor Tissue

Classification was performed in MATLAB (2022a, MathWorks). Each pixel in the image cube represents a 6-element spectrum. To visualize the variation between- and within-classes, principal component analysis (PCA) of these spectra was performed. PCA takes an n-dimensional (n-variable) dataset and projects it onto n new principal component (PC) axes such that the first axis describes the most variance in the data, and each subsequent axis describes most of the remaining variance. Since most of the variation in the dataset can be visualized using the first few PCs, the remaining PCs can be dropped/ignored, allowing graphical visualization of the dataset in 2D or 3D. Furthermore, dropping the latter PCs removes small variations within the data, potentially reducing noise and improving the performance of classification algorithms.

Pixels were classified using four commonly used spectral classification methods:^29^ linear discriminant analysis (LDA), k-nearest neighbor algorithms (KNN), neural networks (NN), and spectral angle mapping (SAM).

LDA classifies spectra by finding a linear combination of features that maximizes the separation between classes relative to within-class variance in the feature space. KNN algorithms classify spectra by choosing the most frequent classes of KNN data points in the feature space (k=5). While LDA assumes linear decision boundaries, the KNN algorithm is non-parametric, so makes no assumptions about the shape of the decision boundaries. LDA also assumes variables are Gaussian distributed. NNs perform classification by passing an input vector, in this case a 9-band spectrum, through a series of artificial neurons, with each neuron outputting some non-linear function of its inputs with some weight that is adjusted during training. The output values of the final layer determine the classification. In contrast to LDA, NN classification does not make assumptions about the distribution of input data nor the shape of decision boundaries. The NN was implemented using a 2-layer feed-forward network, with a sigmoid transfer function in the hidden layer (10 neurons) and a linear transfer function in the output layer, using the MATLAB (MathWorks) “neural network pattern recognition app.”

For LDA, KNN and NN classifiers, the 6-element spectra in the training and test datasets were projected onto PCs determined from the training dataset prior to training/testing the classifier (“PCA-LDA,” “PCA-KNN,” and “PCA-NN,” respectively). The effect of dropping low-variance PCs was also investigated by retaining only the first 4, 5, or 6 PCs for classification. For NN, the 6-element spectra were also tested without projection onto PCs.

SAM calculates the n-dimensional spectral angle (SA) between a target spectrum and a reference spectrum; in this case n=6. The reference spectra are the mean spectra per-class within the training dataset; thus 3 spectral angles are calculated for each target spectrum — $\theta_{\text{tumor}}$, $\theta_{\text{non-tumor-tissue}}$, and $\theta_{\text{background}}$. For a simple SAM classification, the minimum of these three angles is taken as the predicted class (SAM minimum angle). Alternatively, the angles may be treated as 3-element feature vectors and classified using LDA or KNN (SA-LDA and SA-KNN, respectively).

In summary, seven classification methods were compared: PCA-LDA, PCA-KNN, SA minimum angle, SAM-LDA, SAM-KNN, NN, and PCA-NN. Classification accuracy was determined using cross-validation, with each of the image cubes being used for training and the remaining three image cubes being used for testing (four permutations). For NN classification, one image cube was used for training, one image cube was used for validation, and two image cubes were used for testing. Presented classification accuracies represent the average over all permutations.

### 2.6 Simulating the Effects of Exposure on Classification Accuracy

In the real world, FGS imaging conditions vary considerably [Fig. 1(a)]. Many of these variations, such as changes in working distance, illumination intensity and exposure time, can be summarized as a multiplicative change in the light intensity reaching the detector. In the present study, these changes are collectively referred to as changes of “exposure.” To investigate whether classification approaches are robust to changes in exposure, image cubes were multiplied by an exposure factor, E, prior to classification (E=1 is equivalent to the un-modified image cubes used to train the classifiers).

### 2.7 Comparing Multispectral and Monochromatic Fluorescence Imaging

To compare multispectral fluorescence imaging to standard monochromatic fluorescence imaging (fluorescence imaging using a single emission filter), classification of a single filter image (640  pixels×512  pixels×1 filter) was compared with classification based on an image cube (640  pixels×512  pixels×6 filters).

## 3 Results

### 3.1 SWIR Fluorescence Imaging Enables High Tumor-to-Background Ratio

**Fig. 3:**
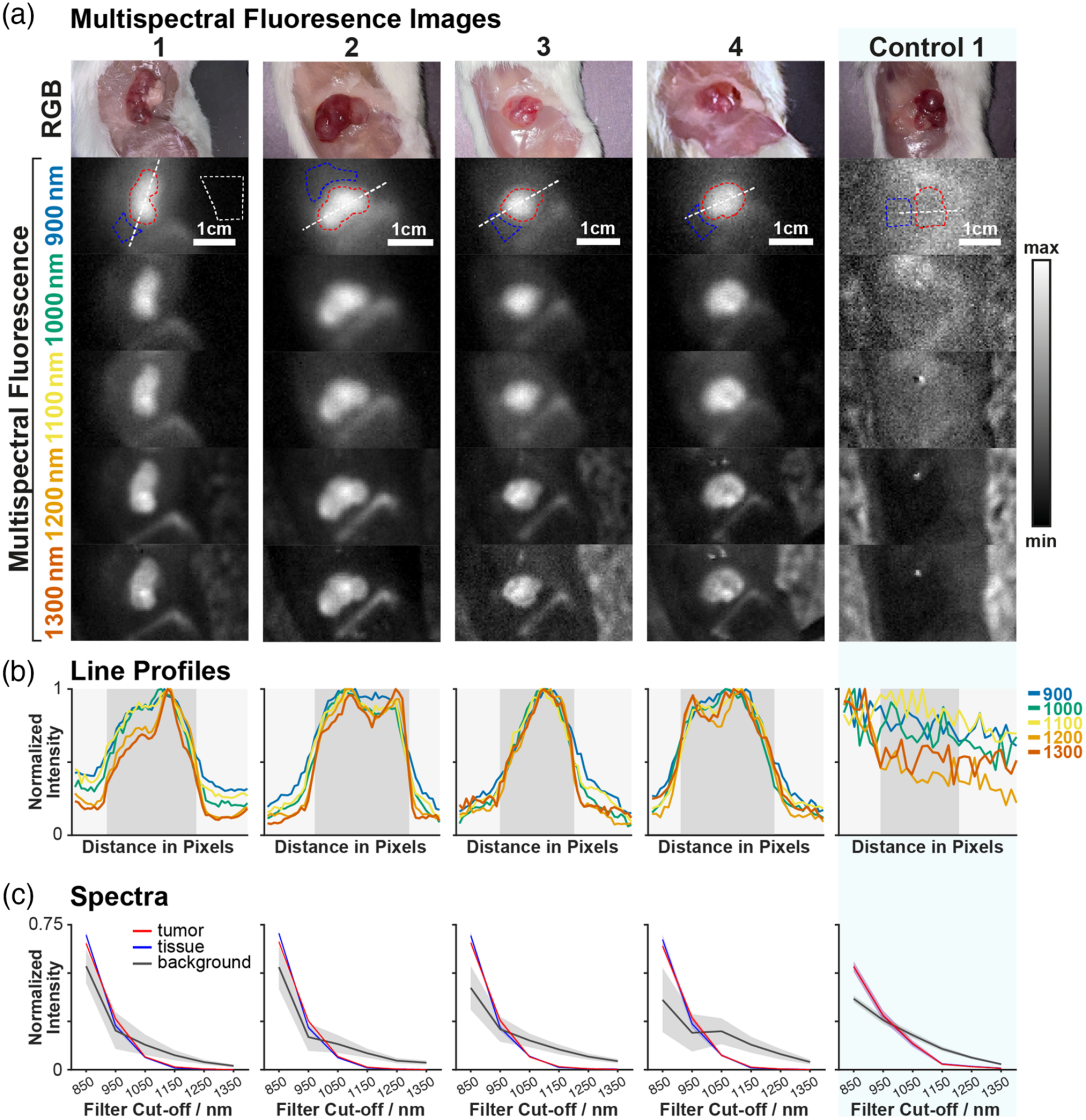
(a) Red-green-blue (RGB) and max-normalized fluorescence band images of tumors in four Dinutuximab-IRDye800 injected mice and one representative tumor-bearing control mouse not injected with the dye. Dashed red line is tumor ROI. Dashed blue line is non-tumor tissue ROI. Dashed white line is the background ROI (shown only for mouse 1 [24h]). (b) Line profiles of fluorescence intensity across the tumor for each wavelength band. The gray region shows the tumor region. (c) Mean spectra from within the ROIs. The shaded areas represent the standard deviation over pixels within each ROI.

To investigate SWIR FGS, a multispectral SWIR fluorescence imaging device was constructed and deployed in a preclinical study. Four mice with subcutaneous NB xenografts were imaged at 24, 48, 72, and 96 h (individuals 1–4, respectively) following the injection of Dinutuximab-IRDye800 and 2 control mice were imaged without Dinutuximanb-IRDye800. The acquired images enabled the construction of band images representing fluorescence collected from 100 nm acceptance bands centered at 900, 1000, 1100, 1200, and 1300 nm (850–950, 950–1050, 1050–1150, 1150–1250, and 1250–1350 nm), allowing the relationship between contrast and wavelength to be investigated (Fig. 3).

The controls show negligible fluorescence intensity (control versus Dinutuximab-IRDye800; 19±5 versus 960±320 at >850  nm), confirming a lack of autofluorescence in the SWIR region. In the Dinutuximab-IRDye800 injected individuals, signal is observed in the non-tumor tissue region, suggesting off-target binding, and scattering of both the on-target and off-target fluorescence.

### 3.2 SWIR Fluorescence Imaging Enables Deep Fluorescence Imaging

**Fig. 4:**
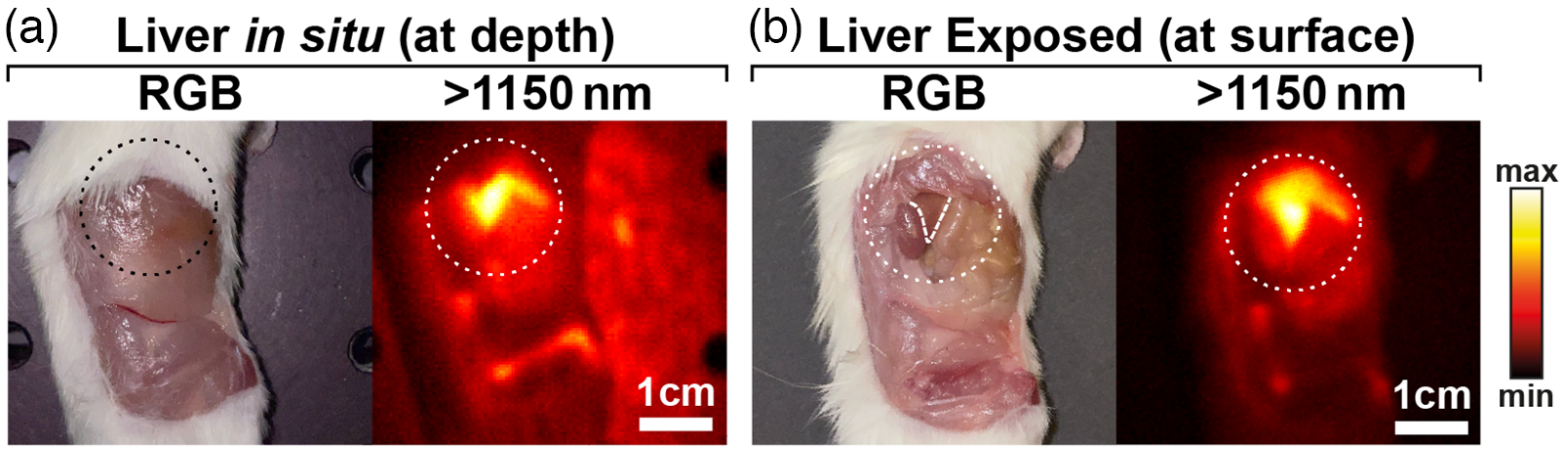
SWIR enables fluorescence imaging at depth. (a) RGB color image and >1150  nm fluorescence image of a mouse following tumor resection. Off-target fluorescence of the liver can be seen beneath the surface. (b) Subsequent exposure of the liver confirms the location and shape of the observed sub-surface fluorescence corresponding to liver tissue.

After tumor resection, one individual was imaged to assess the background in absence of the tumor [Fig. 4(a)]. Off-target liver fluorescence is clearly visible from beneath the tissue surface. Though this is an undesirable off-target effect, it provided an opportunity to observe the depth imaging capabilities of SWIR fluorescence. The liver was surgically exposed to reveal its true location [Fig. 4(b)], confirming the SWIR images accurately delineated the triangular shape of the organ, even as it was buried beneath muscle tissue.

### 3.3 Machine-Learning Combined with Multispectral SWIR Fluorescence Imaging Enables Accurate Tumor Classification

**Fig. 5:**
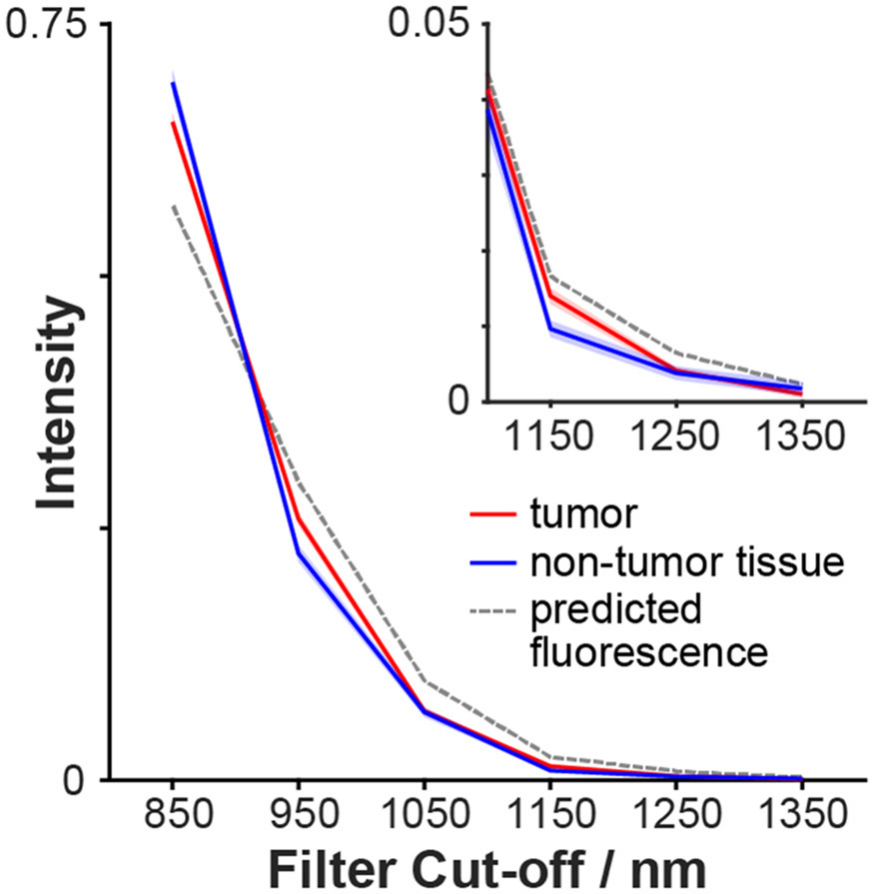
Average spectra of tumor tissue and non-tumor tissue show subtle but conserved differences. The mean spectra from within the tumor and non-tumor ROIs were calculated for each individual. The red and blue lines represent the mean of these spectra over the individuals (n=4), with the shaded regions representing the standard deviation over individuals. The dotted line represents the predicted fluorescence spectrum of pure IRDye800CW.

**Fig. 6:**
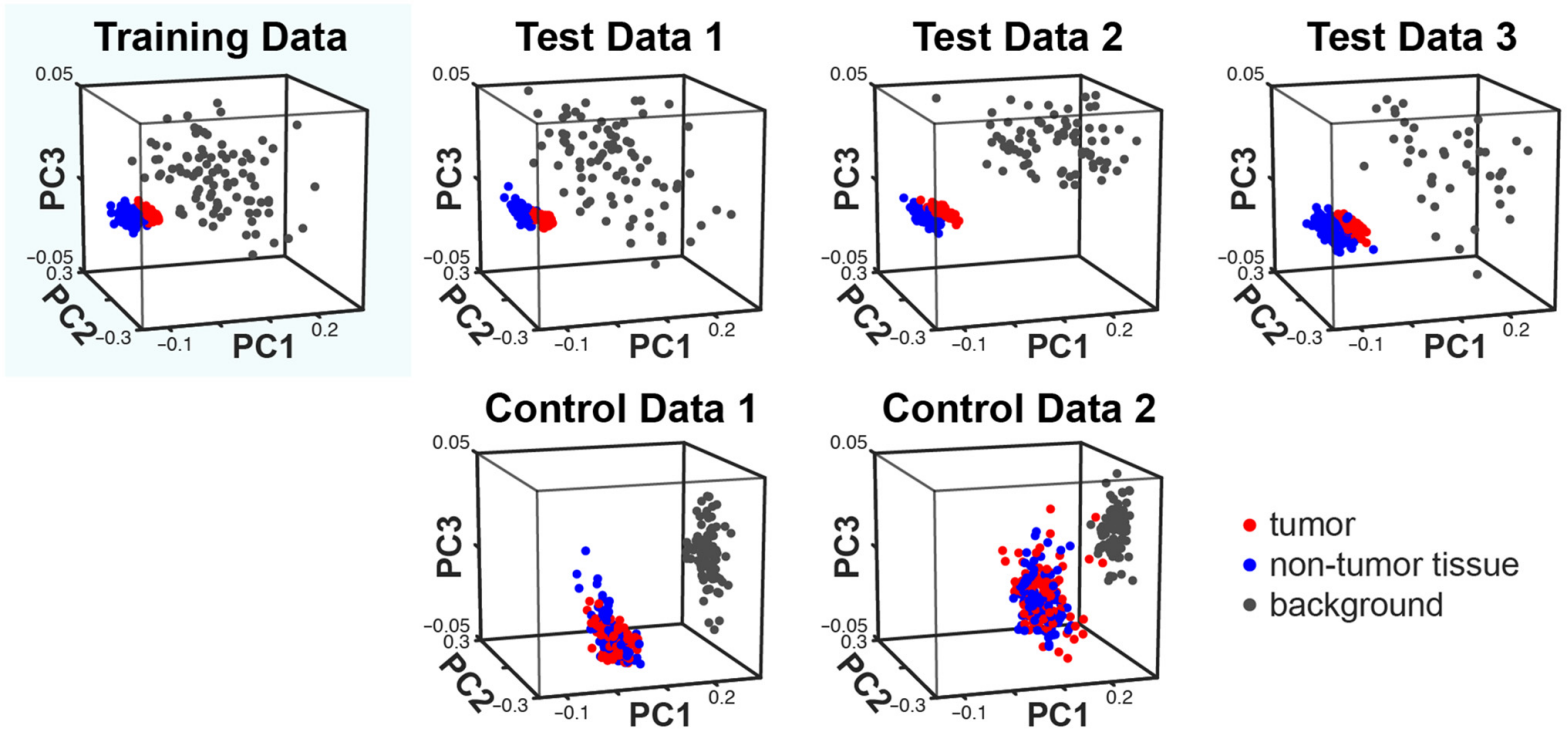
PCA analysis of measured spectra shows distinct conserved clustering for each class. PCA analysis was performed on the spectra from the mouse 1 (24 h). Spectra from the remaining individuals were projected onto the PC axes of mouse 1. Distinct clusters can be observed. The controls do not show any spectra in the region where tumor and non-tumor tissue clusters appear in the fluorescence-injected/non-control individuals.

We hypothesized that fluorescence emission reaching the detector via scattering in non-tumor tissue would have a different spectrum to fluorescence emission arriving directly from the tumor. To test this, average spectra from within tumor and non-tumor tissue ROIs were plotted [Fig. 3(c)]. Indeed, the spectra are distinct and conserved across individuals (Fig. 5). PCA analysis was performed on spectra from mouse 1 (24 h), then spectra from the remaining mice were projected onto the mouse 1 PCs. The spectra clearly cluster by class, with this clustering conserved across the individuals (Fig. 6).

**Fig. 7:**
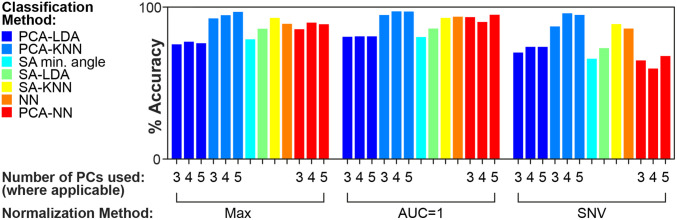
Comparison of seven approaches used for classification of spectra. The methods are described in Sec. 2.4. For each approach, three different normalization methods were used: max normalization, division by the max value; AUC = 1 normalization; and SNV normalization. Classification accuracy was determined using cross-validation. KNN, k-nearest neighbor algorithm; LDA, linear discriminant analysis; NN, neural network; PC, principal component; PCA, principal component analysis; and SA, spectral angle.

**Fig. 8:**
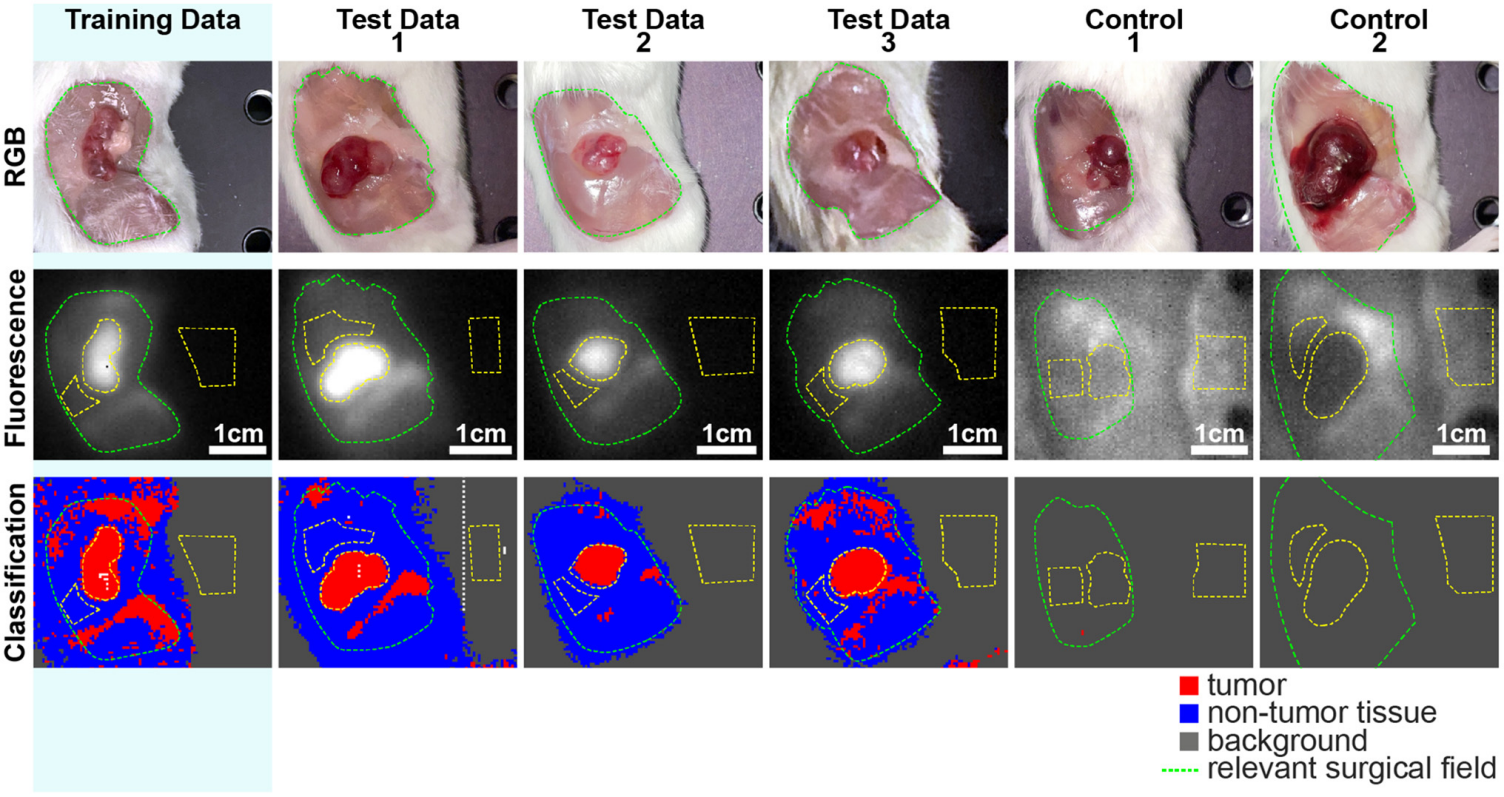
Machine-learning enables classification of multispectral SWIR fluorescence images with high accuracy. Classification maps for PCA-KNN with AUC=1 normalization and 4 PCs. Training data is from mouse 1 (24 h). Test data 1–3 are from individuals 2–4 (48, 72, and 96 h).

Classification accuracy was determined using cross-validation, with 1 image cube being used for training and three image cubes being used for testing (four permutations). The ROIs drawn in each image cube contained 954, 886, 984, and 1060 pixel-spectra for individuals 1–4, respectively. Seven classification methods and three normalization approaches were compared (Fig. 7). The best performing method was PCA-KNN with AUC=1 normalization using 4 PCs. Classification was possible with 97.5% accuracy (97.1%, 93.5%, and 99.2% accuracy for tumor, non-tumor tissue and background, respectively). Confusion matrices for this method are shown in Fig. S2 in the Supplementary Material. Classification maps are shown in Fig. 8. Though there is some misclassification around off-target sources of fluorescence (femur and liver), the tumor is well delineated from the surrounding healthy tissue.

### 3.4 Multispectral SWIR Fluorescence Imaging Enables Exposure-Independent Tumor Delineation

**Fig. 9:**
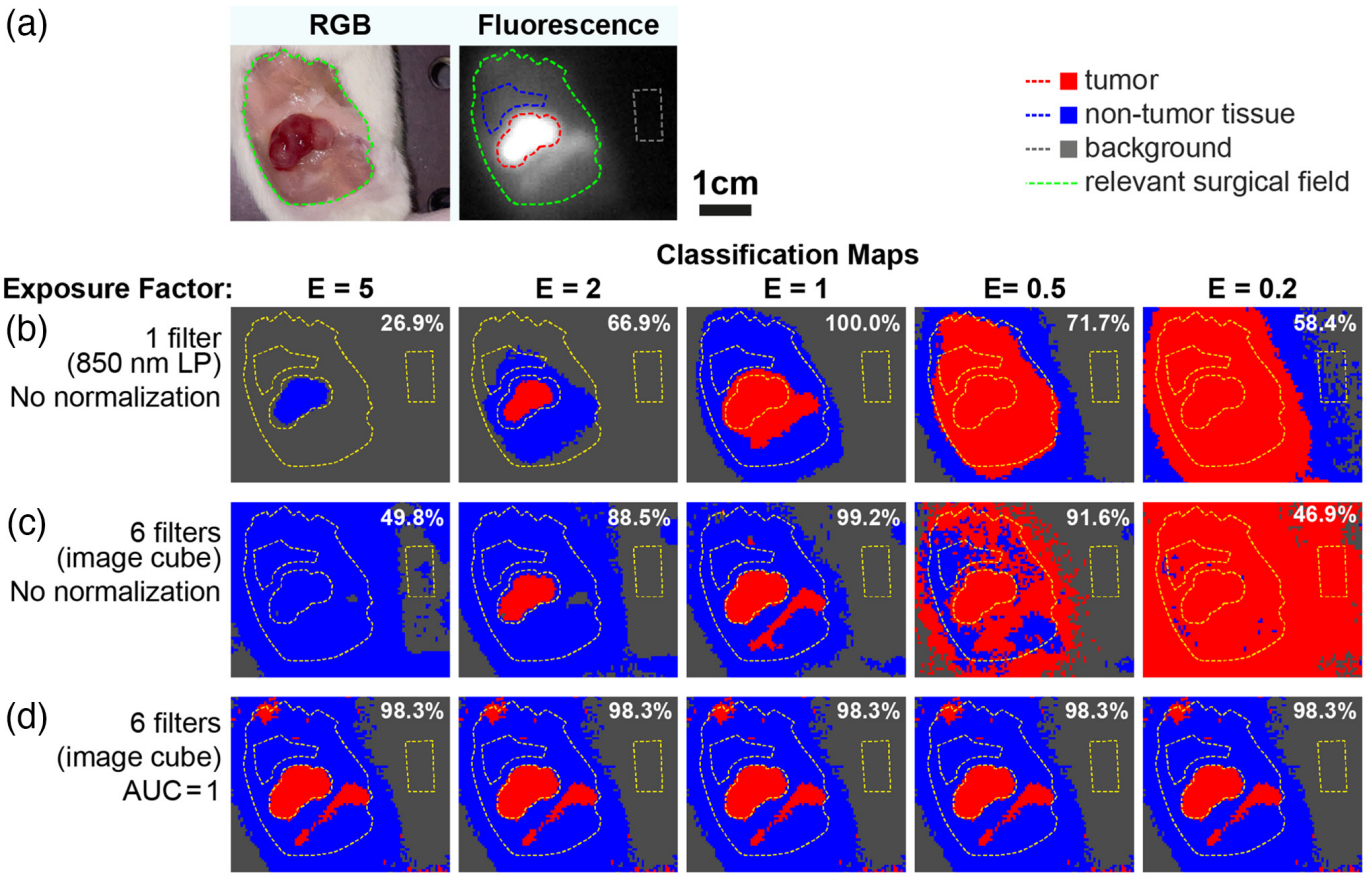
Multispectral SWIR fluorescence imaging enables exposure-factor-independent tumor delineation. (a) RGB color and fluorescence images of the tumor. (b) Classification with just 1 filter image (monochromatic fluorescence image), changes in exposure factor cause large changes in classification maps. Normalization is not possible with a single wavelength. (c) With a 6-filter image cube, but no normalization, classification is more robust to changes in exposure factor, but still fails to properly demarcate the tumor. (d) With both a 6-filter image cube and AUC = 1 normalization, classification is unaffected by changes in exposure factor (by definition). All classification maps are generated using PCA-KNN (k=5 neighbors, PCs=1 in B, PCs = 4 in (c) and (d) trained on mouse 1 (24 h) and tested on mouse 2 (48 h). E=1 corresponds to the exposure factor of the training data. Accuracy over all pixels within the three ROIs is shown in the top right corner of each classification map.

Standard FGS uses a monochromatic fluorescence image captured with a single emission filter. Since any classification based on this image must use an imaging-condition-dependent threshold, the resulting classification is susceptible to errors when imaging conditions change. This can be seen in images captured with different exposures and thresholded to show fluorescence overlays (Fig. S3 in the Supplementary Material). If the exposure time is changed, the apparent size of the tumor changes, resulting in false positives at higher exposures / lower thresholds and false negatives at lower exposures / higher thresholds. Even with machine-learning techniques, classification based on an image acquired with a single emission filter (850-nm LP) is highly susceptible to errors due to changes in the exposure factor [Fig. 9(b)], as these methods ultimately use a threshold to define the class boundaries, albeit one determined statistically based on the training data.

Classification based on a multispectral image cube results in some improvement to classification accuracy, but since the major difference between classes remains their absolute intensity (rather than spectral shape), classifiers still rely primarily on intensity information, and consequently, changes in exposure factor continue to cause large errors in classification [Fig. 9(c)]. By normalizing the pixel-spectra (AUC=1), classification is based only on spectral shape (not absolute intensity). Thus, classification accuracy is independent of exposure factor (as the exposure factor is divided out during pixel-spectra normalization), demonstrating the potential of multispectral fluorescence imaging for robust fluorescence delineation in real-world FGS [Fig. 9(d)].

In summary, robust tumor delineation requires multispectral information and appropriate normalization; classification approaches applied to monochromatic images fail, since normalization of these images is not possible (without tricky calibration) [Fig. 9(b)], and classification of multispectral images fails without normalization [Fig. 9(c)].

## 4 Discussion

FGS is a game-changing innovation with the potential to revolutionize tumor resection by providing surgeons with molecular-level insight into the surgical field. Dozens of targeted fluorescent agents are reaching the end of early-phase clinical trials.^3^ Despite the avalanche of potential new dyes, hardware for FGS has seen little innovation beyond superficial improvements to resolution and display—functional capabilities have seen little change in the past decade. Now is the time to couple new dyes with cutting-edge imaging technology to reach clinical impact.

While FGS works well in preclinical imaging studies, where imaging conditions are carefully controlled, and equipment is well calibrated, the dynamic environment of the operating theatre poses further challenges. Many external factors affect the magnitude of the measured fluorescence intensity, so defining the threshold for detection is difficult. We hypothesized that multispectral FGS would enable image segmentation based on spectral information, rather than intensity information, and thus enable more robust delineation of tumor tissue during FGS.

To test this hypothesis, we constructed a multispectral SWIR fluorescence imaging device capable of acquiring a 6-channel image cube from ∼850 to 1450 nm. This device was deployed in a preclinical imaging study to acquire multispectral image cubes of NB xenografts injected with Dinutuximab-IRDye800. ROIs were drawn on these image cubes to define regions of tumor, non-tumor tissue and background. The tumor and non-tumor tissue spectra were distinct; though the differences were subtle, they were conserved across individuals, facilitating the training of a generalizable classifier.

Seven classification methods, each with three normalization approaches, were trained to classify pixels as tumor, non-tumor tissue, or background based on their spectra. PCA-KNN with AUC=1 normalization using 4 PCs was found to provide the best performance in our dataset, classifying with 97.5% accuracy (97.1%, 93.5%, and 99.2% accuracy for tumor, non-tumor tissue, and background, respectively). Moreover, since classification used spectra normalized to AUC=1, the results do not depend on the absolute intensity of fluorescence, suggesting the classification is robust to changes in imaging conditions that affect the exposure factor. If these results are validated in a first-in-human pilot study, multispectral SWIR fluorescence imaging could be incorporated into clinical practice to improve FGS.

Beyond the merits displayed in the present study, multispectral FGS has the potential to allow spectral unmixing of surgical or background lights; identification and removal of specular reflections; spectral unmixing of autofluorescence;^30^ measurement of tissue absorption to enable non-invasive measurement of oxygenation saturation^31^ or lipid content;^20^ and multiplexing of multiple fluorescent probes.^31^^,^^32^ Exploiting these opportunities will be the objective of future work.

While the results of this study are very promising, this first experience of applying multispectral SWIR fluorescence imaging revealed several limitations that will inform future work. First, our imaging device was constructed using a manual filter wheel, which meant acquiring an image cube was slow. This also limited the number of individuals we could image, resulting in a small cohort of mice. A second-generation system should employ an alternative method of multispectral imaging than enables higher temporal resolution. The present study revealed that differences between tumor and non-tumor spectra are subtle, so future systems might also employ higher spectral resolution, or optimized spectral filter sets specifically designed to distinguish between these spectra.^33^

Second, SWIR imaging required long exposure times (∼2000  ms typical) compared to NIR-I imaging (∼50  ms typical) due to the low emission of the NIR-I dyes in the long wavelength region. For clinical SWIR imaging, short exposure times are desirable to enable video-rate imaging, so future work is required to optimize illumination, field of view, lenses, and filters for an intraoperative SWIR platform.

A third limitation is the use of manually drawn ROIs as ground truth. This did not prove problematic in the current study, but as fluorescence-based tumor delineation becomes more precise, the ground truth position of the margin must likewise become more precise to enable proper assessment of accuracy. Typically, ground truth requires histopathological assessment of tumor margins, but the disparate scales of microscopy and fluorescence imaging make co-registration challenging. *Ex vivo* tissue sections can be imaged microscopically and macroscopically, thus allowing the tumor boundary to be correlated with the presence of fluorescence. However, this is not useful in spectral imaging, where the bulk tissue cannot be omitted due to its optical effect on the spectra. Careful consideration of these challenges should be made in future studies.

In summary, by combining the merits of (i) the long SWIR emission tail of IRDye800CW, (ii) the SWIR sensitivity of InGaAs sensors, (iii) multispectral imaging, and (iv) machine-learning techniques, multispectral SWIR fluorescence imaging demonstrated 97.5% accuracy per-pixel for classifying tumor tissue in a preclinical model of NB. With further development, multispectral SWIR FGS has the potential to revolutionise surgery. The imminent arrival of dozens of new imaging agents provides a timely opportunity for this technology—by enhancing the performance of these agents, multispectral SWIR FGS is poised to be instrumental to the advancement of FGS into the next generation.

## Code, Data, and Materials Availability

Data underlying the results presented in this paper will be made available upon publication.
