# Supplementary material for: Enhancing intraoperative tumor delineation with multispectral short-wave infrared fluorescence imaging and machine learning
Source: J Biomed Opt. 2023 Mar 27;28(9):094804. doi: 10.1117/1.JBO.28.9.094804 (PMC10042297; doi:10.1117/1.JBO.28.9.094804)
Supplement: Supplementary file 1 [file JBO_028_094804_SD001.pdf]

Supplementary Materials

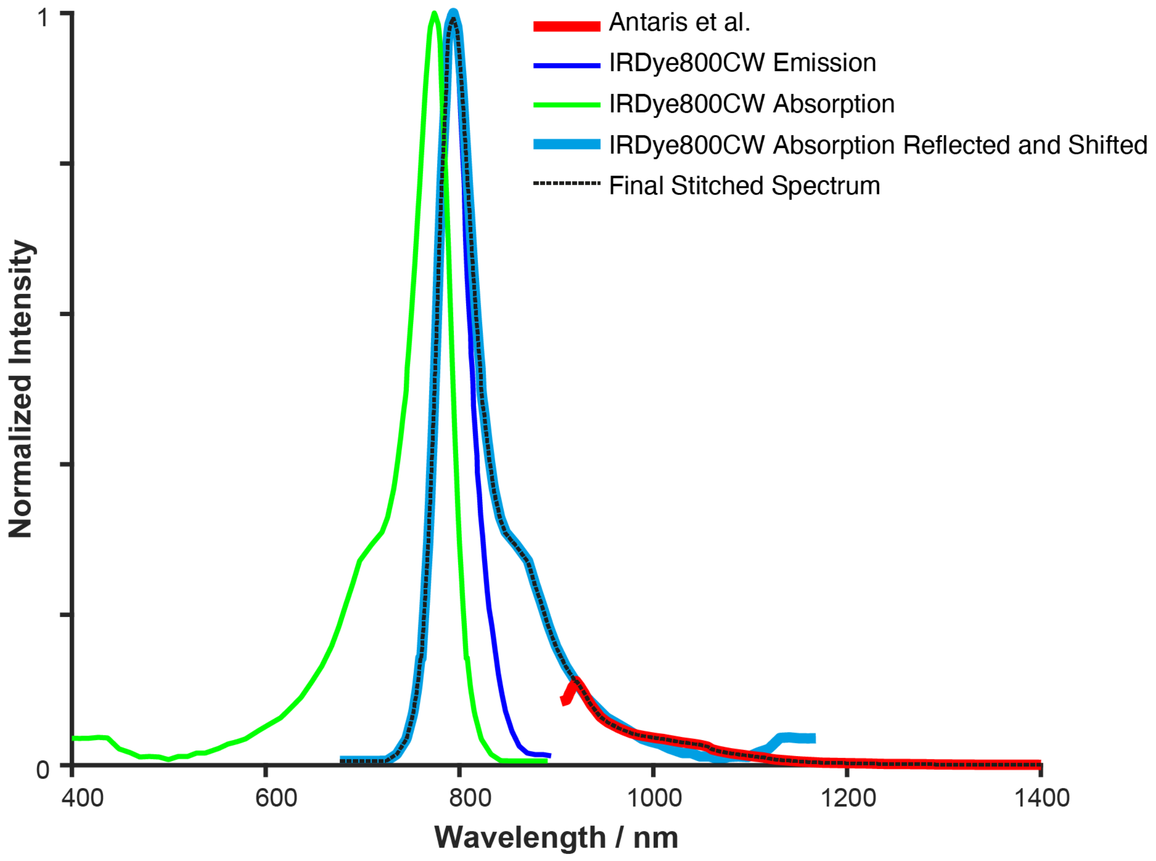

Figure S1. The composed emission spectrum of IRDye800CW (LICOR, USA). IRDye800CW emission at >920nm (red line) adapted from Antaris et al.[10]. IRDye800CW absorption and emission spectra (green and dark blue lines respectively) from LICOR website[23]. IRDye800CW spectrum <920nm was derived by reflecting the IRDye800CW absorption spectrum (Franck-Condon principle) and shifting to align the peak with the peak of the data book IRDye800CW emission spectrum. The scale was then adjusted manually to match the Antaris et al.'s spectrum at 920nm.

True Class \ Predicted Class	A: Tumour	A: Non-Tumour Tissue	A: Background	B: Tumour	B: Non-Tumour Tissue	B: Background	C: Tumour	C: Non-Tumour Tissue	C: Background	D: Tumour	D: Non-Tumour Tissue	D: Background
Tumour	986	69	0	1011	14	0	1077	32	0	1052	10	0
Non-Tumour Tissue	11	482	0	27	335	0	58	444	0	25	469	0
Background	0	0	1382	1	0	1610	17	1	1271	18	6	1244

Figure S2. Confusion matrices for PCA-KNN, AUC=1 normalization, number of PCs = 4. The confusion matrices **A-D** are from the test dataset with training data from mice 1–4 respectively.

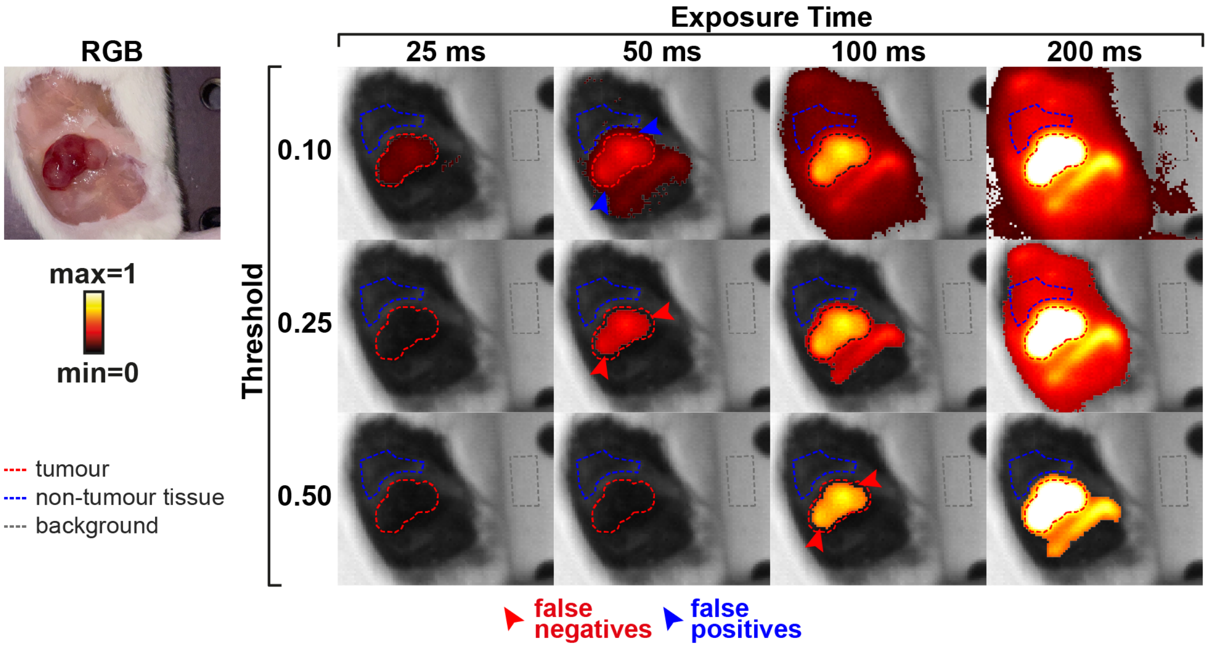

Figure S3. Fluorescence images captured at different exposure times overlaid on a monochrome image with different thresholds. Changing threshold and exposure time changes the apparent size (and shape) of the tumour region, resulting in false positives and false negatives (some of which are shown with blue and red arrows respectively). Fluorescence images acquired with 1150nm long pass filter.
